# Modified Electroconvulsive Therapy in a Patient with Gastric Adenocarcinoma and Metastases to Bone and Liver

**DOI:** 10.1155/2014/203910

**Published:** 2014-09-16

**Authors:** Gennie Wang, Brian Milne, Rachel Rooney, Tarit Saha

**Affiliations:** ^1^School of Medicine, Queen's University, 80 Barrie Street, Kingston, ON, Canada K7L 3N6; ^2^Department of Anesthesiology & Perioperative Medicine, Queen's University, Victory 2, Kingston General Hospital, 76 Stuart Street, Kingston, ON, Canada K7L 2V7

## Abstract

*Background*. In addition to general anesthesia, muscle relaxants are given prior to electroconvulsive therapy (ECT) in order to prevent musculoskeletal injury. Higher doses of muscle relaxants have been suggested for patients at high risk for bone fractures; however, there are adverse side effects associated with these higher doses. *Aims*. We present a successful case of ECT to treat chronic major depressive disorder in a 62-year-old woman at high risk of bone fracture due to gastric adenocarcinoma with metastases to bone and liver. *Case*. Increasing doses of the muscle relaxant succinylcholine (0.45–0.74 mg/kg) were sufficient to prevent musculoskeletal complications throughout the course of 9 bifrontal ECT treatments. Following treatment, the patient reported and demonstrated markedly improved mood and functionality, enabling her transfer to a palliative care facility. *Conclusion*. Standard doses of succinylcholine were sufficient to mitigate the risk of pathological fractures in this patient with metastatic bone lesions. As there are established risks to using high doses of succinylcholine, with no evidence that higher doses reduce the incidence of fractures in high-risk populations, we suggest taking a conservative approach, using clinical observation and periodic plain radiography to dictate succinylcholine dose titration in such high-risk patients.

## 1. Introduction

Modified electroconvulsive therapy (ECT) has been recognized as a safe and effective treatment for major depressive disorders [1]. Standard anesthetic techniques involve the use of a rapid, short-acting induction agent for very brief general anesthesia and a muscle relaxant to prevent serious musculoskeletal complications [2]. Succinylcholine is the most commonly used muscle relaxant, with a typical dose of 0.5 to 1 mg/kg [3]. It has been suggested that higher doses may be required for patients who are at increased risk for bone fractures, such as those with preexisting fractures, severe osteoporosis, or other bone diseases [3]. However, there is currently little information on the management of ECT for cancer patients with bone metastases. Beale et al. did report a case of a patient with prostate cancer and metastatic cancer in the right femoral head who received 12 bilateral ECTs. Treatments were well tolerated but no details of anesthesia were provided [4]. It remains unclear whether or not there is a need to use high doses of muscle relaxants in order to mitigate the risk for bone fracture in cancer patients with bone metastases. Given the adverse side effects associated with high doses of muscle relaxants [5–9], it is important to assess whether low doses are sufficient to protect against bone fractures in high-risk patients.

## 2. Case Presentation

Approval for presentation of this anonymous case report was provided by the patient as well as our institutional research ethics board. A 62-year-old, 67.2 kg, female with a diagnosis of gastric adenocarcinoma complicated by a mood disorder secondary to her diagnosis had suffered from chronic major depressive disorder over many years, with a history of suicide attempts. The patient's longstanding chronic major depressive disorder had become refractory to medications including escitalopram, mirtazapine, lorazepam, and quetiapine.

Previously, the patient underwent unilateral ECT on 2 separate occasions with a positive response, which facilitated antidepressant maintenance. Unfortunately, she relapsed both times due to life stressors after several years of effective management. The cancer was diagnosed incidentally upon hospital admission following a suicide attempt. Computed tomography and magnetic resonance imaging revealed multiple lesions on the liver, two spinal lesions (T12, L4) with no tumour-related impingement of the spinal cord, disc protrusions (T6-7, T7-8) with possible malignant changes, a right femoral neck lesion, and significant arthritic degeneration in her back likely contributing to her back pain. A liver biopsy suggested likely gastric primary adenocarcinoma. Given her poorly managed major depressive disorder and positive response to previous ECT treatments, a series of bilateral ECT treatments were scheduled. Pre-ECT workup revealed anemia, elevated serum calcium, and liver dysfunction. Echocardiogram and chest X-rays were unremarkable. Her physical exam was notable for an enlarged liver crossing the midline 3 cm and below the costal margin 8–10 cm. No ascites was evident.

The patient underwent a series of 9 bifrontal ECTs (1 millisecond/30–40 Hz/3-4 second/144–256 mC/25.3–52.3 Joules/stimulus) using a Spectrum 5000Q device (MECTA Corporation). The stimulus dose was calculated based on the half-age method. Anesthesia was achieved with propofol in decreasing doses of 0.89 mg/kg for the first 4 treatments and 0.74 mg/kg for the last 5 treatments; 0.45 *μ*g/kg of remifentanil was given in treatments 2 to 6, and 0.60 *μ*g/kg was given for the last 3 treatments. Succinylcholine was given in escalating doses of 0.45 mg/kg for the first treatment, 0.60 mg/kg for the next 6 treatments, and 0.74 mg/kg for the last 2 treatments for muscle relaxation. The propofol dose was decreased and the succinylcholine dose was titrated upwards in later treatments to lower seizure threshold and to provide increased safety in prevention against fractures based on visual assessment of muscle contractions at the time of ECT.

The ECT treatments were well tolerated by the patient, except for a reported increase in back pain following the sixth treatment. A plain film of the spine was unremarkable for recent spinal compression or fracture. Following the ECT series, the patient reported and demonstrated marked improvement in mood and functionality which allowed for her transfer to a palliative care facility.

## 3. Discussion

Although ECT has been in use since 1938, the current practice of using anesthesia for ECT was implemented much later [2]. Modified ECT, in which the patient is given an induction agent and muscle relaxant, virtually eliminated the risk of fractures and related musculoskeletal complications in the musculoskeletally “normal” patient [10]. Nevertheless, there are still concerns with regard to musculoskeletal injuries in high-risk populations.

There have been reports of fractures occurring in patients with severe osteoporosis, and in the elderly, despite the use of modified ECT administration [11–15]. Some have reported successful and complication-free ECT treatments when complete muscle paralysis was achieved using higher than standard doses of succinylcholine (>1 mg/kg). Such reports included a patient with osteogenesis imperfecta and vertebral compression fractures [16], a patient with Harrington rods [17], and a patient with severe cervical spine disease [18]. However, there have been other reports of successful ECT treatments using only incomplete muscle relaxation, including one patient with Harrington rods [19] and two patients with preexisting bone fractures [20]. In these cases, the authors cite good interdisciplinary cooperation in the prevention of musculoskeletal injuries.

There is a paucity of literature on the safety of ECT in patients with cancer and bone metastases. Beale et al. reported a case of a patient with prostate cancer and metastases to the femur who received 12 bilateral ECT treatments without complications, though details regarding the anesthetic technique were not provided [4]. As there are currently no standards of practice regarding the use and risks of ECT in patients at higher risk of fractures, it is unknown whether it is more appropriate to induce complete muscle relaxation, or if standard doses of a muscle relaxant are adequate to prevent musculoskeletal complications.

The most commonly used muscle relaxant for ECT is succinylcholine. Increasing the dose of succinylcholine is not without potential adverse effects. Although currently controversial due to conflicting data, there have been reports of more severe muscle pain with higher doses of succinylcholine up to a dose of 1.5 mg/kg [5, 6]. Initial muscle fasciculations following higher doses of succinylcholine may also cause additional trauma and increase the risk of fracture. Myalgia is often accompanied by muscle stiffness and can last for several days, causing considerable discomfort to some patients [7]. It has been shown that, at a succinylcholine dose of 3 mg/kg, the incidence of postoperative myalgia is lower than at doses of either 0.5 or 1.5 mg/kg [5]; however, concerns for other side effects make this dosage inadvisable. Increased dosages of succinylcholine have also been associated with increased risk of apnea and increased time to ventilation recovery [8]. Thus, using higher doses of succinylcholine may require the use of ventilation and/or intubation, resulting in longer recovery times and longer hospital stay.

Other potential side effects of increased succinylcholine dosages include cardiac dysrhythmias (bradycardia, ventricular dysrhythmias, and cardiac arrest) [9] and hyperkalemia, particularly in patients with coexisting disease, such as renal failure, spinal cord injury, stroke, or Parkinson's disease [2]. While higher doses of succinylcholine may be associated with reduced fracture risk, higher doses also increase the risk of developing the above-mentioned side effects. Thus, a more conservative approach to dosing may be beneficial in cases such as the one described here. In this case, our decision to initiate and titrate treatments with standard doses of succinylcholine was guided by interdisciplinary cooperation, clinical observation, and periodic plain radiographs. Use of a peripheral nerve stimulator prior to electrical stimulation may have been beneficial for assessing the degree of muscle paralysis in response to succinylcholine in our patient. In a previous case report on a patient at high risk of fractures due to severe osteoporosis, a peripheral nerve stimulator over the ulnar nerve was used to accurately identify the degree of muscle relaxation, which allowed the physician to avoid using more than one succinylcholine dose for multiple ECT treatments per session [21].

Using this relatively conservative approach to anesthesia induction and muscle relaxation, our high fracture risk patient successfully completed 9 ECT treatments with a positive response in mood and functionality with no evidence of fractures. This approach permitted the patient to be stabilized and allowed for her transfer to a palliative care facility.
